# Giant Cystic Pheochromocytoma with Low Risk of Malignancy: A Case Report and Literature Review

**DOI:** 10.1155/2017/4638608

**Published:** 2017-03-15

**Authors:** Ravi Maharaj, Sangeeta Parbhu, Wesley Ramcharan, Shanta Baijoo, Wesley Greaves, Dave Harnanan, Wayne A. Warner

**Affiliations:** ^1^Department of Clinical Surgical Sciences, University of the West Indies, Eric Williams Medical Sciences Complex, Champ Fleurs, Trinidad and Tobago; ^2^Division of Oncology, Siteman Cancer Center, Washington University School of Medicine, St. Louis, MO 63110, USA; ^3^Department of Cell Biology and Physiology, Washington University School of Medicine, St. Louis, MO 63110, USA

## Abstract

Giant pheochromocytomas are rare silent entities that do not present with the classical symptoms commonly seen in catecholamine-secreting tumors. In many cases they are accidentally discovered. The algorithm to diagnose a pheochromocytoma consists of biochemical evaluation and imaging of a retroperitoneal mass. The female patient in this case report presented with a palpable abdominal mass and was cured with surgical resection. She suffered no recurrence or complications on follow-up. The left retroperitoneal mass measured 27 × 18 × 12 cm and weighed 3,315 grams. Biochemical, radiological, and pathological examinations confirmed the diagnosis of a pheochromocytoma. In this paper, we report on our experience treating this patient and provide a summary of all giant pheochromocytomas greater than 10 cm reported to date in English language medical journals. Our patient's giant cystic pheochromocytoma was the fourth heaviest and fifth largest maximal diameter identified using our literature search criteria. Additionally, this tumor had the largest maximal diameter of all histologically confirmed benign/low metastatic risk pheochromocytomas. Giant cystic pheochromocytomas are rare entities requiring clinical suspicion coupled with strategic diagnostic evaluation to confirm the diagnosis.

## 1. Introduction

 A pheochromocytoma (PCC) is a rare catecholamine-secreting tumor that originates from the chromaffin cells of the adrenal medulla. First described by Frankel [1] in 1886, the estimated worldwide incidence of these tumors is 2 to 8 per million persons per year [2]. Classical symptoms at presentation include severe hypertension with associated headaches, sweating, and palpitations; however, 20–30% of patients remain asymptomatic. Asymptomatic PCCs are typically detected as an incidental adrenal mass on routine screening. Biochemical evidence of elevated plasma free metanephrines provides the highest sensitivity for PCC diagnosis [3]. Most of these lesions are benign/low metastatic potential but histopathological characteristics defined by the pheochromocytoma of the adrenal gland scaled score (PASS) can identify tumors with potentially more aggressive biological behavior such as the presence of chromaffin tissue at extra adrenal sites. Giant PCCs are generally classified as those with maximal diameter greater than 10 cm. They are commonly asymptomatic and are diagnosed incidentally on imaging. Surgical resection is the standard treatment option and is usually curative, preventing future potentially lethal complications of these lesions. We present the case of a 50-year-old female patient with a left side adrenal PCC which was treated successfully with open surgical resection in the surgical unit of Eric Williams Medical Sciences Complex, Trinidad and Tobago.

## 2. Case Presentation

A 50-year-old East Indian woman with a 6-month history of lower back pain, fatigue, and unintentional weight loss was referred to our surgical outpatient clinic upon detection of a large abdominal mass during an abdominal ultrasound. The mass was located in the left upper quadrant and thought to be possibly splenic or renal in origin. Physical examination revealed a large nontender lump arising from the left upper quadrant and crossing the midline. Initial laboratory investigations were unremarkable except for microcytic anemia with a mean corpuscular hemoglobin concentration (MCHC) of 7.3 g/dL. The patient had no history of hypertension, headache, palpitations, or excessive sweating and no family history of cancer.

**Figure 1:**
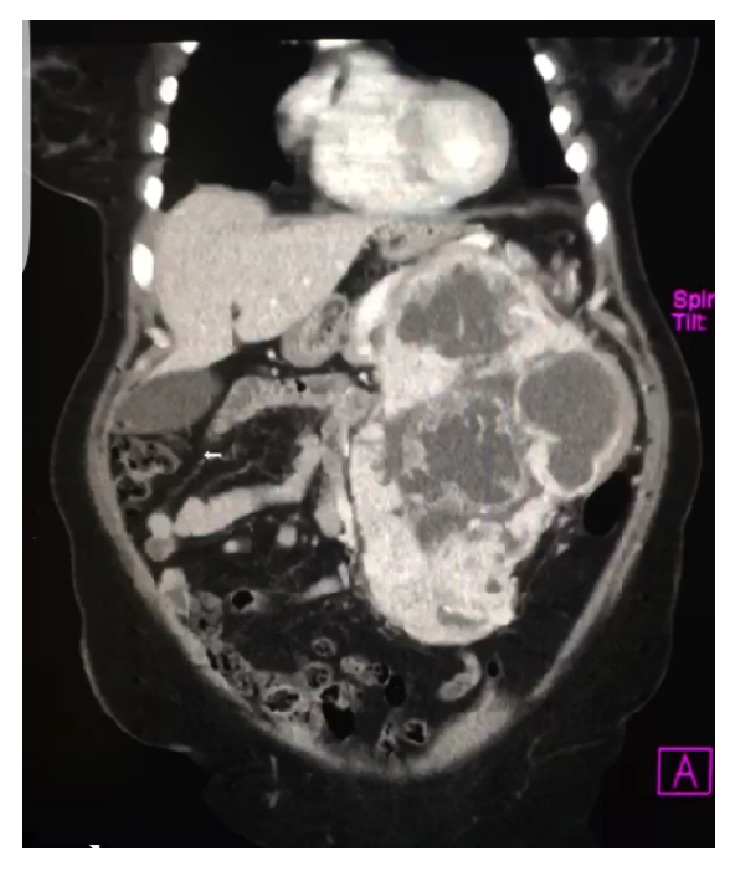
CT scan showing a 23 cm, thick-walled, multicystic mass occupying most of the left upper quadrant of the abdomen.

**Figure 2:**
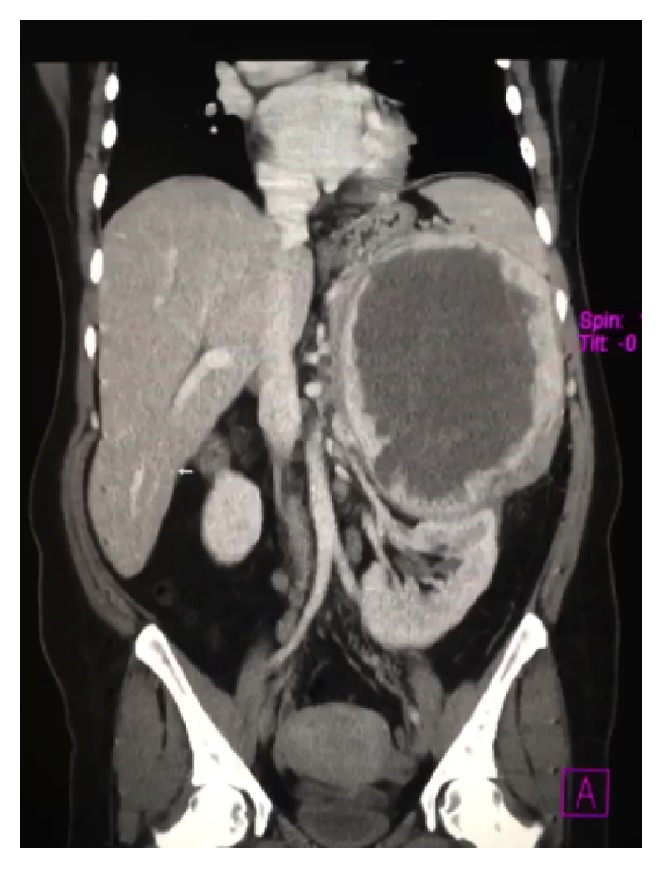
Coronal CT image demonstrating the mass displacing the left kidney inferiorly. Evidence of locally invasive disease was not present.

Computed tomography (CT) revealed a 23.2 × 17.6 × 13.6 cm mass with predominant areas of necrosis, punctate areas of calcification, and irregular contours superior to the left kidney (Figures 1 and 2). The mass displaced the pancreas anteriorly and compressed the left kidney inferiorly. The nonvisualization of a normal left adrenal gland strongly suggested an adrenocortical carcinoma. The liver, spleen, right adrenal gland, and right kidney were normal. Despite mesenteric and para-aortic lymphadenopathy there was no evidence of distant metastasis. Preoperative biopsy was not performed, due to the risk of tumor seeding along the biopsy path.

The tumor markers carcinoembryonic antigen, carbohydrate antigen (CA) 15-3, CA 19-9, and CA 125 were normal. The differential diagnosis included adrenocortical carcinoma, pheochromocytoma, myelolipoma, metastasis from an unidentified primary tumor, sarcoma, and lymphoma. Biochemical investigations were performed to exclude a functional adrenal mass, and the diagnosis of pheochromocytoma was made upon observation of elevated plasma free metanephrines, urine catecholamines, and their metabolites (Table 1).

**Figure 3:**
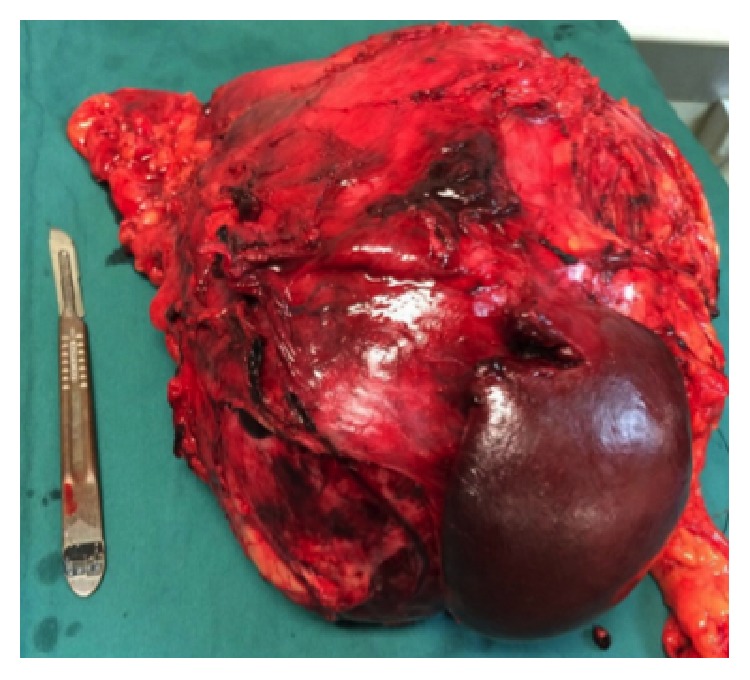
En bloc resection of the left adrenal mass, pancreatic tail, spleen, and left kidney.

Preoperatively, the patient was transfused with packed red blood cells to stable hemoglobin of 10 g/dL. Adequate catecholamine blockade was achieved, after medical consultation using the alpha adrenergic blocker, terazosin (Hytrin). An open left adrenalectomy was performed through a Chevron incision extending to the left flank. The mass was completely resected en bloc with the spleen, distal pancreas, left kidney, and a 2 cm area of the left hemidiaphragm (Figure 3). Intraoperatively, there were significant fluctuations in the patient's blood pressure, which was well managed by the surgical team. There were no other significant surgical complications and the patient made an uneventful recovery prior to discharge on postoperative day 11. Three months later, at the time of this report, the patient remains stable and disease-free. Given that there is recurrence in approximately 10% of these cases, long-term follow-up with CT scans and hematologic monitoring is warranted.

**Figure 4:**
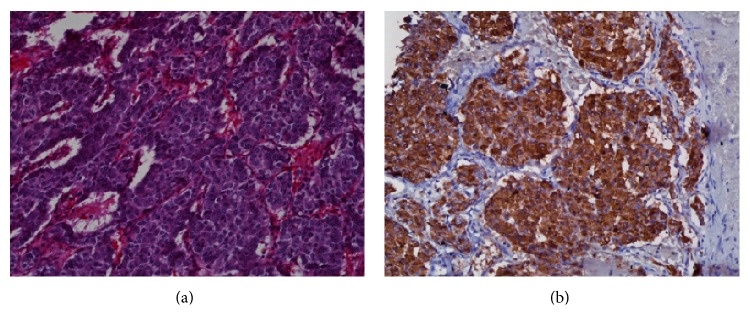
Microscopic images of pheochromocytoma. (a) Routine hematoxylin and eosin (H&E) staining at low magnification (20x) highlights the typical “Zellballen” growth pattern characterized by nests of tumor cells surrounded by delicate fibrovascular stroma. (b) Immunohistochemistry for the neuroendocrine marker chromogranin A shows strong, diffuse staining in the tumor cells.

Pathological examination of the surgical specimen revealed a circumscribed mass, surrounded by a variably thick fibrous capsule/pseudocapsule with relatively scantly attached fat, measuring 27 × 18 × 12 cm and weighing 3,315 g (Figure 3). Residual normal-appearing adrenal gland parenchyma was present, while neither infiltration of the surrounding fat, vascular invasion, nor confluent tumor necrosis was identified. Mitoses were rare (<1 per high-power field). Histological sections revealed typical morphological features of PCC predominantly characterized by nests of plump tumor cells with abundant basophilic granular cytoplasm surrounded by sustentacular cells (Figure 4(a)). No unfavorable features such as diffuse growth pattern, increased cellularity, or increased pleomorphism were observed. The spleen, distal pancreas, and kidney did not show any pathologic abnormalities. Immunohistochemical staining revealed that the tumor cells were diffusely positive for chromogranin A (Figure 4(b)), while there was no significant highlighting of sustentacular cells by S-100. Of note, the lack of prominent staining of sustentacular cells by S-100 has been suggested to be predictive of nonfamilial sporadic PCC [38].

To determine the clinicopathological features of giant PCCs, an extensive literature search of the PubMed/MEDLINE and Embase databases was conducted. Search terms included “giant pheochromocytoma”, “cystic giant pheochromocytoma”, “English language” and “case reports”. One case in which the tumor lacked dimensions but was found to be one of the heaviest PCCs recorded was included. Full texts were accessed to confirm eligibility for inclusion. A summary of the 36 final cases are presented in Table 2. Relative to the others, our patient's tumor was the fourth heaviest with the fifth greatest maximal diameter and the largest histologically confirmed pheochromocytoma with a low risk of malignancy/benign classification. Unlike patients with classical symptoms, those with giant PCCs may be asymptomatic as occurred in 31% (=11) of the cases. Twenty-two % (=8) and 17% (=6) of the cases presented with hypertension and back/abdominal pain, respectively. Only one case had evidence of metastasis at the time of diagnosis, characterized by invasion of the right lobe of the liver [5]. Twenty-two percent (=8) of the cases presented at similar locations, commonly in the left abdomen. The mean age at diagnosis was 49.46 years (range 12–85 years) which was the age of our patient.

## 3. Discussion

Pheochromocytomas are neoplasms that arise from the chromaffin cells of the sympathoadrenal system [39]. Eighty-five percent of these lesions arise in the adrenal medulla [40]. Sporadic cases of PCC usually present in the fourth to fifth decades of life. Various hereditary conditions such as multiple endocrine neoplasia 2A and 2B, Von Hippel-Lindau syndrome, and neurofibromatosis 1 are associated with increased risk for PCC [41]. There are no known environmental, dietary, or lifestyle risk factors that impact the risk of developing PCC. The classic tetrad of symptoms consists of palpitations, headaches, sweating, and hypertension. However, approximately 49–57% of PCC patients are asymptomatic with an adrenal mass being detected incidentally during unrelated imaging.

The malignant potential of a PCC cannot be determined preoperatively unless there is evidence of local invasion or metastases at the time of diagnosis. Previously, it was believed that size was a predictor of malignant potential; however, with numerous case reports of giant benign PCCs, size can no longer be used as a definitive indicator of aggressive disease [42]. Histologically, the PASS is referenced to distinguish tumors of high from those with a low risk of malignancy [43]. This scoring system assesses vascular invasion, capsular invasion, extension into the periadrenal adipose tissue, the presence of focal or confluent necrosis, high cellularity, tumor cell spindling, cellular monotony, >3 mitoses per high-power field, atypical mitotic figures, profound nuclear pleomorphism, and increased tumor cell hyperchromasia [43]. Biologically aggressive tumors have been found to have a PASS ≥ 4 whereas lesions with a low risk of malignancy have a PASS < 4. Our patient harbored a giant PCC with a low risk of malignancy as noted by the PASS of 2.

Approximately 20 to 30% of all PCCs are clinically silent [12]. Factors that contribute to the lack of symptoms include extensive necrosis of the adrenal gland, decreasing the production of catecholamines and the retention of these hormones within the capsular mass after secretion. Consequently, the time to diagnosis is delayed and tumor size tends to be larger once it is detected. A recent report that reviewed 20 cases of PCCs larger than 10 cm reported that 13 presented with abdominal pain with only 5 presenting with any of the classical symptoms of PCCs [16].

Surgical resection is the only curative option for these giant lesions. Laparoscopic adrenalectomy is considered safe and effective for tumors up to 12 cm in its greatest dimensions. However, in the realm of these giant PCCs, open en bloc resection is required. Intraoperative manipulation of these tumors is frequently associated with profound hypertension. However, early isolation of the tumor's venous drainage decreases the risk of intraoperative hypertensive crises [36]. This must be coupled with catecholamine blockade and intravenous fluids to diminish the risk of postoperative hypotension. It is therefore critical to have good coordination between the anesthesiologist and surgeon before and during surgery. Intensive care monitoring is crucial for at least 24 hours postoperatively as it is common for patients to experience fluctuations in blood pressure and heart rate as well as hypoglycemia. Pheochromocytomas have an excellent prognosis, with 5-year survival exceeding 95% in benign tumors and a recurrence rate of less than 10% [44]. Statistical data are not available for malignant PCCs due to their low incidence.

## 4. Conclusion

In this report, we presented the case of a 50-year-old female with a giant PCC that was the fourth heaviest and had the fifth largest maximal diameter of all reported PCCs. Additionally, using our search criteria, the tumor had the largest maximal diameter of reported, histologically confirmed PCCs with a low risk of malignancy. Our patient, 3 months after resection of the tumor, remains stable and disease-free. Giant PCCs do not present with the classical symptoms associated with smaller PCCs and are usually associated with a lower risk of malignancy. Previously, larger size was believed to be an indicator of malignancy in these adrenal lesions; however, upon review of the 10 largest known PCCs, this belief was unsubstantiated.

## Figures and Tables

**Table 1:** Biochemical investigations confirming the pheochromocytoma diagnosis.

	Patient values	Normal values
*Test (fractionated plasma)*		
Metanephrine	259 pg/mL	<58 pg/mL
Normetanephrine	4603 pg/mL	<149 pg/mL
Total metanephrine	4862 pg/mL	<206 pg/mL
Epinephrine	16 pg/mL	<84 pg/mL
Norepinephrine	509 pg/mL	<420 pg/mL
Dopamine	<30 pg/mL	<60 pg/mL
Total catecholamines	525 pg/mL	<504 pg/mL
*Test (24-hour urine values)*		
Total catecholamines	131 ug/24 hr	<100 ug/24 hr
Norepinephrine	120 ug/24 hr	<80 ug/24 hr
Epinephrine	11 ug/24 hr	<20 ug/24 hr
Dopamine	466 ug/24 hr	<500 ug/24 hr
Vanillylmandelate (VMA)	88.3 ug/24 hr	3.8–6.7 mg/24 hr
Creatinine	1.47 ug/24 hr	0.63–2.50 g/24 hr

**Table 2:** A summary of reported giant pheochromocytomas with maximal diameter greater than 10 cm, arranged by largest to smallest maximum diameter. The weight was not recorded in many of the papers.

Author/year	Sex/age	Country	Size (cm)/weight (g)	Location	Presentation	Histopathological evaluation	Metastasis
Grissom et al./1979 [4]	F/54	USA	45 × 25/3000+	Left abdomen	Asymptomatic	Unknown	None
Costa et al./2008 [5]	M/46	Brazil	30/unknown	Right adrenal	Abdominal pain	Malignant	Liver
Basso et al./1996 [6]	M/47	Italy	29 × 21 × 12/4050	Left abdomen	Asymptomatic	Malignant	None
Karumanchery et al./2012 [7]	F/85	England	28 × 16 × 13/2300	Left abdomen	Lower back pain	Unknown	None
Current case	F/50	Trinidad & Tobago	27 × 18 × 12/3315	Left abdomen	Lower back pain	Low risk of malignancy	None
Gupta et al./2016 [8]	F/65	India	25 × 17 × 15/2750	Left abdomen	Asymptomatic	Benign	None
Okuda et al./2013 [9]	F/43	Japan	24 × 23 × 16/5900	Abdomen	Vulva edema	Unknown	None
Suga et al./2000 [10]	M/48	Japan	21 × 13 × 21/3900	Left abdomen	Asymptomatic	Unknown	None
Terk et al./1993 [11]	M/35	USA	21 × 20 × 11/2870	Organ of Zuckerkandl	Disproportionate abdominal girth, hypertension	Unknown	None
Soufi et al./2012 [12]	F/17	India	21 × 15	Right upper abdomen	Asymptomatic	Malignant	None
Arcos et al./2009 [13]	F/36	Canada	21 × 17 × 11	Left abdomen	Lower back pain	Malignant	Lymph node
Melegh et al./2002 [14]	M/55	Hungary	20/unknown	Left renal hilus	Asymptomatic	Unknown	None
Korgali et al./2014 [15]	M/63	Turkey	20 × 17 × 9/1736	Left adrenal gland	Chest pain, sweating, nausea	Malignant	Rib
Ambati et al./2014 [16]	F/77	Canada	19 × 18 × 12/2460	Right retroperitoneum	Dyspnea	Benign	None
Pan et al./2008 [17]	M/46	USA	18 × 14 × 13/1450	Left abdomen	Episodic hypertension and headache	Unknown	Unknown
Uysal et al./2015 [18]	M/37	Turkey	18 × 8 × 13	Left abdomen	Hypertension	Malignant	Multiorgan^*∗*^
Sharma/2006 [19]	M/55	India	17 × 12/850	Right adrenal gland	Asymptomatic	Benign	None
Daughtry et al./1977 [20]	M/53	USA	17/1150	Unknown	Mild hypertension	Unknown	Unknown
Gupta et al./2016 [8]	M/40	India	16.4 × 14 /1836	Left thyroid gland	Severe hypertension	Unknown	Unknown
Costa et al./2008 [5]	F/43	Brazil	16	Right upper abdomen	Abdominal pain	Malignant	Unknown
Jain and Agarwal /2002 [21]	F/26	India	16 × 11	Left abdomen	Asymptomatic	Malignant	Unknown
Wu et al./2000 [22]	F/49	USA	15 × 12 × 12	Right upper abdomen	Asymptomatic	Unknown	Unknown
Santarone et al./2008 [23]	F/81	Italy	13	Right upper abdomen	Hypertension, palpitation, sweating	Unknown	Unknown
Sundahl et al./2016 [24]	F/54	Sweden	12.5 × 10 × 3/204	Right adrenal gland	Asymptomatic	Benign	None
Zhu et al./2014 [25]	F/67	Japan	12.1 × 10.8	Left adrenal gland	Dizziness, vomiting, and stomachache	Unknown	None
Ikegami et al./2009 [26]	M/47	Japan	12 × 10	Abdomen	Back pain	Unknown	Unknown
Li et al./2012 [27]	M/56	Canada	12 × 11 × 11	Right upper abdomen	Progressive weight loss and nausea	Benign	None
Kakoki et al./2015 [28]	M/70	Japan	12 × 11 × 8/530	Left adrenal gland	Ileus after hypertension medication	Benign	None
Schnakenburg et al./1976 [29]	M/12	Ukraine	12 × 10 × 9/1100	Right abdomen	Hemihypertrophy	Malignant	Lung, brain
Filippou et al./2003 [30]	M/70	Greece	12 × 8 × 10	Left abdomen	Asymptomatic	Malignant	None
Sarveswaran et al./2015 [31]	F/59	India	11.2 × 9.6 × 9.8	Right suprarenal region	Upper right abdominal discomfort	Unknown	Unknown
Chan et al./2000 [32]	M/63	China	11 × 6.6 × 11	Right suprarenal region	Asymptomatic	Malignant	Bone, lung
Awada et al./2003 [33]	F/26	USA	11 × 10 × 9	Right adrenal gland	Dyspnea, paresthesia, chest pain, palpitation	Unknown	Unknown
Goldberg et al./2011 [34]	F/27	Canada	10.5 × 10.6	Right adrenal gland region	Headaches, episodic palpitations, pallor	Unknown	Unknown
Antedomenico and Wascher/2005 [35]	F/39	USA	10.5/782	Left upper abdomen	Abdominal pain	Unknown	Unknown
Wang et al./2015 [36]	F/36	China	10.3 × 9.3	Left upper abdomen	Abdominal pain	Unknown	Unknown
Basiri and Radfar/2010 [37]	M/53	Iran	Unknown/3150	Left abdomen	Abdominal pain	Benign	None

M, male; F, female. ^*∗*^Liver, lymph nodes, right adrenal gland, lungs, and bones.
